# Binding free energies for the SAMPL8 CB8 “Drugs of Abuse” challenge from umbrella sampling combined with Hamiltonian replica exchange

**DOI:** 10.1007/s10822-021-00439-w

**Published:** 2022-01-03

**Authors:** Daniel Markthaler, Hamzeh Kraus, Niels Hansen

**Affiliations:** Institute of Thermodynamics and Thermal Process Engineering, University of Stuttgart, 70569 Stuttgart, Germany

**Keywords:** SAMPL8, Umbrella sampling, Host–guest complex

## Abstract

Umbrella sampling along a one-dimensional order parameter in combination with Hamiltonian replica exchange was employed to calculate the binding free energy of five guest molecules with known affinity to cucurbit[8]uril. A simple empirical approach correcting for the overestimation of the affinity by the GAFF force field was proposed and subsequently applied to the seven guest molecules of the “Drugs of Abuse” SAMPL8 challenge. Compared to the uncorrected binding free energies, the systematic error decreased but quantitative agreement with experiment was only reached for a few compounds. From a retrospective analysis a weak point of the correction term was identified.

**Supplementary Information:**

The online version of this article contains supplementary material available 10.1007/s10822-021-00439-w.

## Introduction

Among the many possible methodological variants to compute binding free energies for host-guest systems within a rigorous thermodynamic framework [1], umbrella sampling (US) [2] has emerged as an easy-to-use approach that is supported by many publicly available pre- and post-analysis tools [3]. If certain artefacts related to insufficient sampling are accounted for [4], the method yields robust estimates. Based on our previous experience with this approach in the context of cyclodextrin host-guest systems [4], we aimed for the evaluation of the simulation protocol in the SAMPL8 challenge, featuring more complex guest molecules than considered before. We hope that the comparison to other free-energy methods applied to the same host-guest systems within the SAMPL challenge is of use for the continuous evaluation of methods and force fields pushing forward the field of binding free energy calculations, which, for complex systems, often faces a combination of force-field and sampling issues [5, 6].

## Computational approach

### Molecular model

**Fig. 1:**
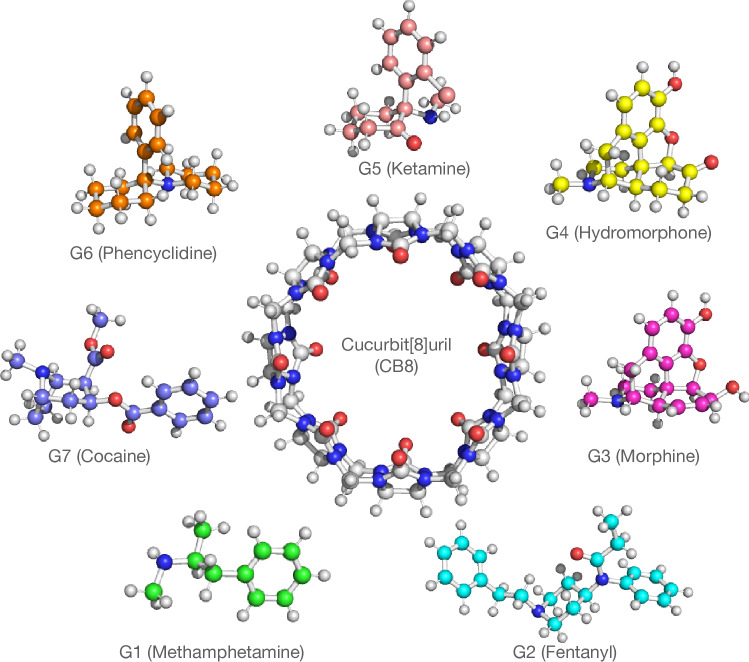
Structures of the CB8 host and the seven guest molecules forming the SAMPL8 “Drugs of Abuse” challenge

Coordinates of host and guest molecules (see Fig. 1) for the SAMPL8 CB8 challenge were obtained from the SAMPL8 GitHub repository [7] in form of mol2 files. In order to incorporate pH effects in the simulation protocol, for every ligand a deprotonated and corresponding protonated form was parametrized. The deprotonated species were created by manually removing the respective hydrogen atoms from the structure files. To generate the topology files, general Amber force field (GAFF) [8] atom types were assigned using the antechamber module of Ambertools20 [9] with the molecule structures as an input. For the protonated types, the partial charges in the provided mol2 file were retained while in the deprotonated cases these were obtained from the semiempirical AM1-BCC model [10, 11].

**Fig. 2:**
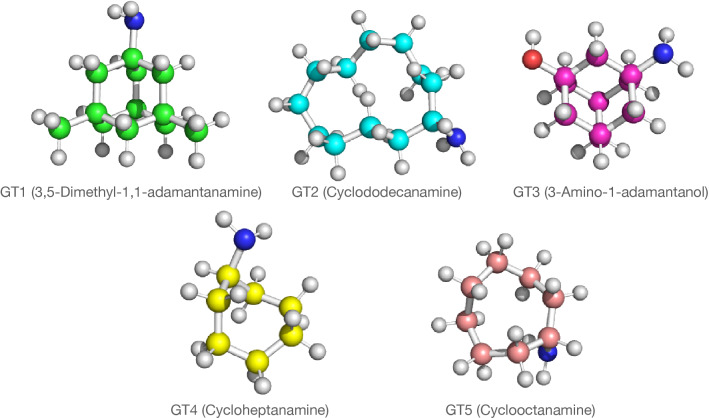
Structures of the training set molecules GT1 to GT5

Due to slight rounding errors in the calculated values, the resulting excess charge had to be distributed among the different atoms in a weighted manner, which was done using the Python community package ParmEd [12]. These molecule files were then used to generate topology files in Amber format using the leap module of Ambertools before converting them into the GROMACS format using the ParmEd package. The same pipeline was used to parametrize the CB7 host and a further set of five guest molecules used for training purposes (see Fig. 2).

The mol2 file of CB7 was obtained from the Amber tutorials website [13]. The additional five guest molecules were 3,5-dimethyl-1,1-adamantanamine (GT1), cyclododecanamine (GT2), 3-amino-1-adamantan-1-ol (GT3), cycloheptanamine (GT4) and cyclooctanamine (GT5). Except for GT1, these molecules were part of the SAMPL6 challenge and the mol2 files were obtained from the corresponding GitHub repository. For GT1, initial coordinates were obtained by DFT optimization [14–16] and the molecular model was parametrized as described above. Water was represented by the TIP3P model [17] while the chloride ion used to neutralize the simulation box was described with the parameters of Joung and Cheatham [18].

Alternative partial charges for the guest molecules were calculated with the DDEC6 approach [19]. The effect on binding free energies was marginal for GT1 and more significant for G1 (compare Fig. S1), but far below the discrepancy between simulation results and experiment. Therefore, all further results correspond to the AM1-BCC partial charges.

### System preparation

For the given concentration of sodium phosphate buffer of 20 mM [20] we estimated the concentration of ${{\mathrm {H}_{\mathrm {2}}}{{\mathrm {PO}}_4^-}}$ and ${{\mathrm {HPO}}_4^{2-}}$ at pH 7.4 by means of the Henderson-Hasselbalch equation [21] using an available online tool [22], resulting in concentrations of 5.40 mM and 14.60 mM, respectively. Although AMBER/GAFF-compatible parameters have been reported for these ions [23], the low concentration (approximately 1 or 2.5 molecules, respectively, per 10000 water molecules) suggests that not capturing the ionic strength is a rather secondary effect for this dataset. Moreover, low ion concentrations may cause sampling issues [24, 25]. Therefore, we decided to include only one $\mathrm {Cl}^-$ counterion for the protonated guest molecules and lumped a possible error caused by not accounting for the ionic strength into a correction anyway applied to the raw MD data (see below).

Available p$K_a$-values for the seven ligands suggest that the protonated form of the molecule is the dominant one in a solution of pH 7.4, except for guest 5, which has a p$K_a$-value of 7.5, and therefore the concentration of the protonated form is only marginally larger than that of the unprotonated form. For all guest molecules both the protonated and deprotonated form were considered in the binding free energy calculations. Reported is the value of the form with higher binding affinity, which was always the protonated form.

### Molecular dynamics simulations

All simulations were conducted with the GROMACS 2016.4 program [26] patched to the free-energy library PLUMED 2.4.2 [27] for restraints definition. Free-energy profiles were constructed from the time series of a single order parameter sampled via umbrella sampling [2]. The order parameter is given by the projection of the instantaneous separation vector between the centers of mass (COM) of the binding partners onto the host’s instantaneous symmetry axis and was sampled between $-2$ and $+2$ nm in steps of 0.1 nm and using a force constant of $4000\,{{\mathrm {kJ\,mol}}^{-1}{\mathrm {nm}}^{-2}}$ for keeping the ligand in the respective umbrella window. Lateral movement of the ligand at every umbrella window was restricted with the aid of a flat-bottom potential acting on the orthogonal displacement of the ligand’s COM from the host’s molecular axis. The flat-bottom width of 0.5 nm was chosen such that no force was acting on the bound ligand. The host’s orientation was aligned along the *z*-axis of the simulation box alongside with a translational restraint ($500\,{{\mathrm {kJ}}\,{\mathrm {mol}}^{-1}{\mathrm {nm}}^{-2}}$) to keep its COM close to the box center. The order parameter was written to file every 100 steps. To facilitate configurational sampling, a Hamiltonian replica exchange scheme [28, 29] was used that attempts an exchange move between neighboring umbrella windows every 1000 steps.

Each sampling window included a minimum of 40 ns simulation time. Each system was solvated with $\sim$ 2000 TIP3P waters in an orthorhombic box whose dimensions were approximately $36 \times 36 \times 52\,{\mathrm {\AA }}^3$. Starting configurations for each umbrella window were generated by displacing the ligand out of the host’s binding pocket through application of the set of restraining potentials described above and simulating for 100 ps in order to relax the system. The bound state of the ligand was determined in a preceding step by allowing the ligand to be captured into the CB cavity within a 2 ns NPT simulation via a harmonic distance restraint (force constant: $2000\,{{\mathrm {kJ}}\,{\mathrm {mol}}^{-1}{\mathrm {nm}}^{-2}}$) applied for the COM-COM distance. The stability of the complex configuration found this way was then further examined within another 2 ns NPT simulation without any restraining potential between ligand and host. This procedure was conducted 3 times within 3 independent simulations and the final snapshots of the bound state configurations were visually compared. In none of the studied cases involving CB8, significant differences could be determined in the 3 respective complex configurations and all bound states were stable within 2 ns unbiased simulations.

Production simulations were conducted in the NPT ensemble at 300 K and ambient pressure, with temperature control using a Langevin thermostat [30] with an inverse friction constant of $\tau _\mathrm {T} = 1.0~\mathrm {ps}$ and Parrinello-Rahman barostat [31, 32] with coupling constant $\tau _\mathrm {p} = 2.0\,\mathrm {ps}$. A Verlet-buffered neighbor list [33] which was updated every 10 steps, was applied for the treatment of short-range electrostatic and van der Waals interactions with potentials shifted to zero at 0.9 nm. The latter were modeled by the Lennard-Jones potential. Analytic dispersion corrections were applied for energy and pressure calculation. Long-range electrostatic interactions were treated with the smooth particle-mesh Ewald (PME) method [34, 35] using a real-space cut-off of 0.9 nm with a cubic splines interpolation scheme and a grid spacing of 0.1 nm. The center of mass translation of the computational box was removed every 100 steps. All bond lengths involving hydrogens were constrained using the SHAKE algorithm [36] with a relative tolerance of 0.0001.

The hydration free energy and octanol water partition coefficient were calculated for the neutral form of G1 from alchemical decoupling of the molecule from the surrounding bulk phase consisting of cubic boxes with one guest molecule in 1000 water molecules or in a mixture of 200 water and 800 1-octanol molecules, respectively, the latter described by the GAFF-DC parameters [37]. The scaling of the non-bonded interactions between the ligand and its environment was controlled via a coupling parameter $\lambda$, such that $\lambda = 0$ and $\lambda = 1$ represents the fully interacting and fully decoupled ligand, respectively, while retaining the intramolecular interactions. The decoupling was conducted in a sequence of 20 discrete steps, using simulations times of 10 ns per $\lambda$-state. In the applied perturbation scheme, electrostatic interactions were deactivated first within 5 steps, followed by the deactivation of the Lennard-Jones interactions. To avoid numerical problems close to the end states, soft-core (sc) potentials were used with parameters $\alpha _\mathrm {sc} = 0.5$, $\sigma _{sc} = 0.3$ nm and a power for the soft-core scaling function of $p_\mathrm {sc}$ = 1 [26]. The free energy changes were estimated from the sampled potential energy differences between all $\lambda$-states using the MBAR estimator [38] as implemented in a freely available Python program [3].

### Analysis

Free energy profiles were analyzed using the umbrella integration (UI) method [39–41] and compared to results from the weighted histogram analysis method (WHAM) [42–44] which showed no substantial differences.

If a free energy offset between the two flat bulk water regions of the free energy profiles was present, it was found to be relatively small (below $8~{{\mathrm {kJ}}\,{\mathrm {mol}}^{-1}}$) compared to the global well depth in all cases, except for the largest ligand G2 ($\approx 35\,{{\mathrm {kJ}}\,{\mathrm {mol}}^{-1}}$), probably pointing towards non-sufficient sampling. In cases for which the offset exceeded the thermal noise threshold ($= RT$), binding free energies were calculated from free energy profiles estimated with the modified WHAM algorithm of Hub et al. [45], using an additional constraint to suppress such offsets, noting that this is an ad-hoc solution that may lead to deviation from the standard binding free energy obtained from an offset-free free energy profile [4]. The standard binding free energy was obtained from the well depth of the free energy profile after correcting for the orthogonal restraint and the standard state concentration as described previously [4, 46, 47].

### Empirical correction

From the results of previous SAMPL-challenges [48] and from own test calculations using guest molecules of known binding affinity, it became evident that the used force field overestimates binding substantially. This was addressed in some SAMPL6 contributions by a correction term of the form

1 $$\begin{aligned} \varDelta G_{\mathrm {corr}} = a \varDelta G_{\mathrm {calc}} + b \end{aligned}$$

where the slope and offset coefficients (i.e., *a* and *b* respectively) were trained on data generated for previous rounds of the challenge [48]. While this correction could reduce the RMSE, it did not appreciably impact correlation statistics [48]. Therefore, an alternative approach was considered in the present work. The raw binding free energy values were corrected by an additive term such that

2 $$\begin{aligned} \varDelta G_{\mathrm {pred}}^{\mathrm {corr}} = \varDelta G_{\mathrm {sim}}^{\mathrm {raw}} - \varDelta G_{\mathrm {corr}} \end{aligned}$$

The functional form of the (possibly very approximate) correction term $\varDelta G_{\mathrm {corr}}$ was chosen to be similar to the expression for the binding free energy in the Linear Interaction Energy approach [49], comprising a contribution from van der Waals interactions and one associated with electrostatic interactions, approximated by a simple descriptor,

3 $$\begin{aligned} \varDelta G_{\mathrm {corr}} = N_{\mathrm {heavy}}\left( \frac{V_{\mathrm {hg}}^{\mathrm {vdW}}}{\varDelta \mathrm {SASA}}\right) \cdot \alpha + {{\mathrm {TPSA}}\cdot \beta } \end{aligned}$$

In this equation, $N_{\mathrm {heavy}}$ denotes the number of heavy atoms that interact with the host molecule, $V_{\mathrm {hg}}^{\mathrm {vdW}}$ represents the average short-range LJ interactions between host and guest in the minimum of the free-energy profile and $\varDelta \mathrm {SASA}$ is the change in solvent accessible surface area upon binding. For the set of five training molecules the ratio $V_{\mathrm {hg}}^{\mathrm {vdW}} / \varDelta \mathrm {SASA}$ turned out to be almost constant. TPSA describes the topological polar surface area obtained from PubChem while $\alpha$ and $\beta$ are fit parameters, which were determined based on a set of five guest molecules with known binding affinity towards CB8. Since the training set comprised relatively small molecules that fit well into the CB8 cavity, the above expression was slightly altered for the set of seven drug molecules such that an effective number of heavy atoms was defined by

4 $$\begin{aligned} N_{\mathrm {heavy}}^{\mathrm {eff}}=\frac{V_{\mathrm {hg}}^{\mathrm {vdW}}}{\langle V_{\mathrm {hg}}^{\mathrm {vdW}} / N_{\mathrm {heavy}} \rangle _{\mathrm {training}}} \end{aligned}$$

where the denominator represents the average short-range LJ interactions per heavy atom for the training set and equals $-2.94\,{{\mathrm {kJ}}\,{\mathrm {mol}}^{-1}\,{\mathrm {atom}}^{-1}}$.

For the set of five training molecules the contribution of the first term to the total correction in Eq. (3) was 97.6%, 81.2%, 70.7%, 71.6% and 75.3%.

## Results and discussion

An initial assessment of force field and methodology to calculate binding free energies was attempted based on experimental data from the literature regarding the hydration free energy of G1 as well as on binding free energies of G1, G2, G5, G6 and G7 to CB7. Subsequently, the binding free energies of guests GT1 to GT5 to CB8 was calculated and used to parametrize an empirical correction model that was then applied to the SAMPL8 set of guest molecules.

### Solvation free energies

For the neutral form of G1 (methamphetamine) a hydration free energy of $-20.2\,{{\mathrm {kJ}}\,{\mathrm {mol}}^{-1}}$ was calculated. Because experimental values were not available directly, estimates were obtained from the relation [50]

5 $$\begin{aligned} c_{\mathrm {G1}}^{\mathrm {s}}=\left( \frac{p_{\mathrm {G1}}^{\mathrm {sat}}}{p^o}\right) \exp \left[ \frac{-\varDelta G_{\mathrm {G1}}^{\mathrm {hyd}}}{RT} \right] \end{aligned}$$

where $c_{\mathrm {G1}}^{\mathrm {s}}$ is the aqueous solubility expressed in molar concentration, *R* is the universal gas constant, *T* is the temperature, $p_{\mathrm {G1}}^{\mathrm {sat}}$ is the vapor pressure of G1 in equilibrium with pure condensed G1 and $p^o$ is the pressure (24.77 bar) of an ideal gas at 1 molar concentration and 298.15 K. Using a solubility from PubChem of $0.928\,{{\mathrm {g}}\,l^{-1}}$ and different estimates of the vapor pressure ranging from 0.72 Pa (PubChem) to 38 Pa [51], a range of possible hydration free energies was obtained with values between $-14.9$ and $-24.7\,{{\mathrm {kJ}}\,{\mathrm {mol}}^{-1}}$. Other theoretical estimates reported in the literature are $-6.65\,{{\mathrm {kJ}}\,{\mathrm {mol}}^{-1}}$ and $-32.5\,{{\mathrm {kJ}}\,{\mathrm {mol}}^{-1}}$ coming from classical density functional calculation and the estimation program interface (EPI) Suite^TM^, respectively [52]. For the solvation free energy in the hydrated octanol phase a value of $-34.95\,{{\mathrm {kJ}}\,{\mathrm {mol}}^{-1}}$ was calculated in the present work, leading to an octanol-water partition coefficient of 2.6, which compares reasonably well with the value of 2.07 reported on PubChem, which is, however, not an experimental estimate. Due to a lack of experimental data for the other compounds, additional solvation free energy simulations were not conducted.

### Binding free energies of SAMPL8 guests to CB7

**Fig. 3:**
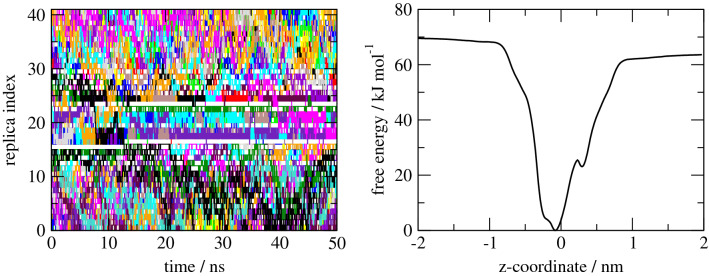
Replica exchanges over simulation time (left) and corresponding free energy profile (right) for the system G1 (protonated form) binding to CB7. Differently colored traces represent different replicas

Binding constants for some guest molecules to CB7 were measured by direct $^1$H NMR titration (G5, G6, G7), $^1$H NMR competition assay (G1) or ITC (G2), respectively [53]. The binding free energy calculations showed some sampling problems at the cavity entrance, illustrated by the gaps in the time series of replica exchanges, see Fig. 3.

For the more bulky guest molecules these sampling issues became more severe such that the simulated binding free energies are likely contaminated by insufficient sampling. The results are reported in Table 1 and show a rather heterogeneous picture that does not allow for an unambiguous assessment of force-field adequacy. The free-energy profiles are shown in the Supplementary Information (see Fig. S2).

**Table 1:** Experimental [53] and simulated binding free energies (${{\mathrm {kJ}}\,{\mathrm {mol}}^{-1}}$) of SAMPL8 guest molecules to CB7. Simulated values are presented for the neutral/protonated form, respectively

Guest molecule	$\varDelta G_{\mathrm {exp}}$	$\varDelta G_{\mathrm {sim}}$
G1 (Methamphetamine)	$-$46.1	$-$35.7 / $-$55.6
G2 (Fentanyl)	$-$41.4	No binding
G3 (Morphine)	No binding	No binding/$-$16.1
G4 (Hydromorphone)	No binding	No binding/$-$17.1
G5 (Ketamine)	$-$16.0	No binding/$-$5.8(R); $-$7.4(S)
G6 (Phencyclidine)	$-$20.8	No binding/$-$22.3
G7 (Cocaine)	$-$19.2	$-$7.5 / $-$44.0

The uncertainty in the simulated values resulting from free-energy profile offsets and statistical errors estimated from fluctuations of the sampled order parameter via error propagation according to the UI method [41] are on the order of $4.2\,{{\mathrm {kJ}}\,{\mathrm {mol}}^{-1}}$. Cases for which no stable bound state (i.e. with the ligand binding either to the inner or outer host surface) could be determined within a series of 3 independent unbiased simulations of 2 ns simulation time, are referred to as as “no binding”

### Binding free energies of training set to CB8

In contrast to CB7, no significant sampling problems at the cavity entrance were observed for the CB8 host. The free-energy profiles of the five training guest molecules are shown in the Supplementary Information (see Fig. S3). In some cases, e.g. GT1, some offset was observed between the two bulk regions. However, compared to the profile well depth this effect is of minor importance.

**Fig. 4:**
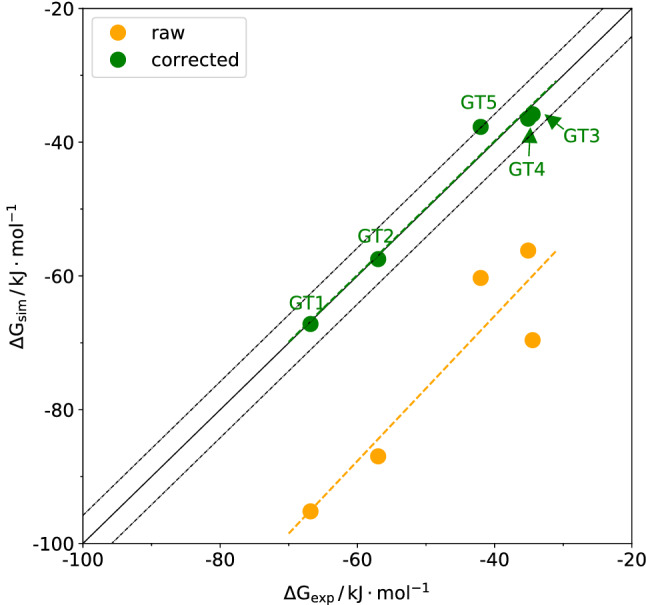
Correlation between calculated and experimental binding free energies for the set of five training molecules with and without an empirical correction. The model parameters required to evaluate Eq. (3) are provided in the Supplementary Information. The fit parameters were $\alpha = 17.8\,{{\mathrm {\AA }}^2}$ and $\beta = -0.21\,{{\mathrm {kJ}}\,{\mathrm {mol}}^{-1}\,{\mathrm {\AA }}^{-2}}$. The uncertainty in the simulated values resulting from free-energy profile offsets and statistical errors estimated from fluctuations of the sampled order parameter via error propagation according to the UI method [41] are on the order of $4.2\,{{\mathrm {kJ}}\,{\mathrm {mol}}^{-1}}$

Fig. 4 shows that the calculated binding free energies correlate well with experiment data but are consistently too negative. By means of Eq. (3), these raw binding free energies were corrected, resulting in an almost perfect agreement with the experimental data. For GT3, GT4 and GT5 the advantage of individualized contributions to the correction term are visible as the corrected data are much less scattered compared to the raw data.

### Binding free energies of SAMPL8 guests to CB8

**Fig. 5:**
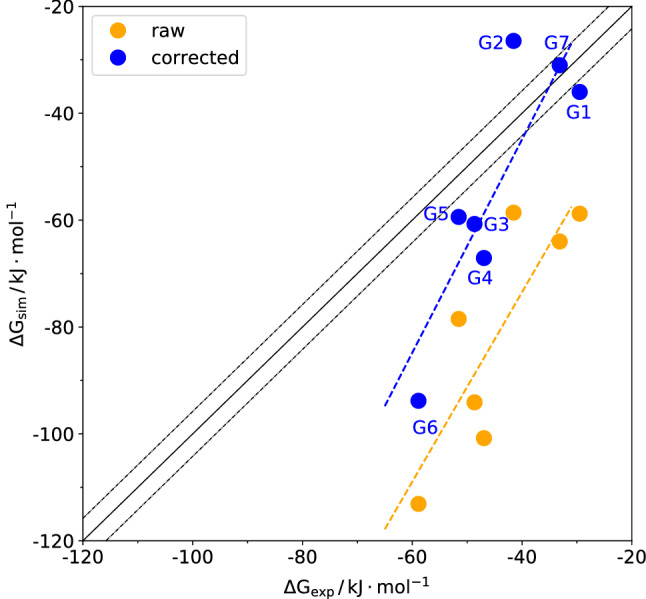
Correlation between calculated and experimental binding free energies for the set of seven challenge molecules with and without an empirical correction. The model parameters required to evaluate Eq. (3) are provided in the Supplementary Information. The fit parameters were $\alpha = 17.8\,\mathrm {\AA ^2}$ and $\beta = -0.21\,{{\mathrm {kJ}}\,{\mathrm {mol}}^{-1}\,{\mathrm {\AA }}^{-2}}$. The uncertainty in the simulated values resulting from free-energy profile offsets and statistical errors estimated from fluctuations of the sampled order parameter via error propagation according to the UI method [41] are on the order of $4.2\,{{\mathrm {kJ}}\,{\mathrm {mol}}^{-1}}$

Free-energy profiles of the seven challenge guest molecules are shown in the Supplementary Information (see Fig. S4). Fig. 5 shows the correlation of raw and corrected binding free energies with experimental values. Note that for the corrected values, Eq. (4) was used to calculate an effective number of heavy atoms, while the parameters $\alpha$ and $\beta$ entering Eq. (3) were transferred from the training set discussed in the previous paragraph. In contrast to the training molecules, the slope of the linear regression curve is larger than one. Unfortunately this slope even gets larger when applying the corrections. Therefore, the systematic error decreased but the statistical correlation did not improve.

**Fig. 6:**
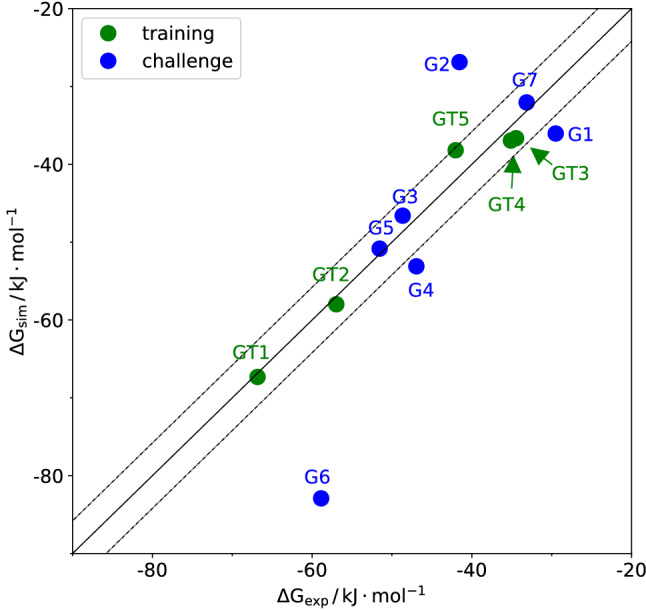
Correlation between calculated and experimental binding free energies for the set of five training molecules and seven challenge molecules after applying Eq. (3) with the actual number of heavy atoms, except for molecules G2 and G7 for which the same effective number was used as in Fig. 5. The fit parameters were $\alpha = 17.8\,{\mathrm {\AA }}^2$ and $\beta = -0.21\,{{\mathrm {kJ}}\,{\mathrm {mol}}^{-1}\,{\mathrm {\AA }}^{-2}}$. The uncertainty in the simulated values resulting from free-energy profile offsets and statistical errors estimated from fluctuations of the sampled order parameter via error propagation according to the UI method [41] are on the order of $4.2\,{{\mathrm {kJ}}\,{\mathrm {mol}}^{-1}}$

If the correction model according to Eq. (3) is re-evaluated using the actual number of heavy atoms instead of the effective number according to Eq. (4), the correlation with experiment improves for those molecules which fit well into the CB8 cavity, i.e. G3, G4 and G5. For G1, the difference between the actual and effective number of heavy atoms is only 0.2. Only for the molecules that do not fit well into the CB8 cavity, i.e. G2 and G7, the evaluation of the correction model with the effective number of heavy atoms leads to better agreement with experiment. For G6, the predicted value gets closer to experiment when using the actual number of heavy atoms but is still far off. The results of this retrospective analysis are displayed in Fig. 6 and may point to an improved correction model for future applications. We also note, that the present results, together with other SAMPL contributions could be re-analysed by efficient reweighting schemes [54] to study the sensitivity of the binding free energy with respect to the various force field parameters.

## Conclusions

Based on previous rounds of the SAMPL challenge in which the GAFF force field overestimated binding affinities to the CB8 host [48], quantitative agreement between simulation and experiment from the raw simulation results was not expected in the present study. An empirical correction model that goes beyond a simple linear correction by incorporating specific properties of the guest molecules as well as of their interactions with the host was proposed. The model worked rather well for the small set of five training molecules. For the seven guest molecules of the SAMPL8 challenge it reduced the systematic error but did not improve the statistical correlation. The good agreement between simulation and experiment obtained by Henchman and co-workers in the SAMPL8 challenge [55] using the GAFF2 force field [9] suggest to reconsider the US simulations in future work to explore the effect of the re-optimized covalent and Lennard-Jones interactions (possibly combined with a revised charge model [56]) relative to the GAFF force field. Umbrella sampling combined with Hamiltonian replica exchange turned out to be a simple and robust simulation protocol, as was also concluded by other participants of the SAMPL8 challenge [57].

## Supplementary Information

Below is the link to the electronic supplementary material.

Electronic supplementary material 1 (PDF 189 kb)

## Data Availability

The datasets generated and analysed in this work are available in the data repository of the University of Stuttgart (DaRUS): https://doi.org/10.18419/darus-2109.
