# Supplementary material for: Binding free energies for the SAMPL8 CB8 “Drugs of Abuse” challenge from umbrella sampling combined with Hamiltonian replica exchange
Source: J Comput Aided Mol Des. 2022 Jan 3;36(1):1–9. doi: 10.1007/s10822-021-00439-w (PMC8831271; doi:10.1007/s10822-021-00439-w)
Supplement: Supplementary file 1 — Electronic supplementary material 1 (PDF 189 kb) [file 10822_2021_439_MOESM1_ESM.pdf]

Supplementary information: Binding free energies for the SAMPL8 CB8 "Drugs of Abuse" challenge from umbrella sampling combined with Hamiltonian replica exchange

Daniel Markthaler, Hamzeh Kraus, and Niels Hansen*

Institute of Thermodynamics and Thermal Process Engineering (ITT), University of Stuttgart, Pfaffenwaldring 9, 70569 Stuttgart, Germany

E-mail: hansen@itt.uni-stuttgart.de

SI1 Free energy profiles

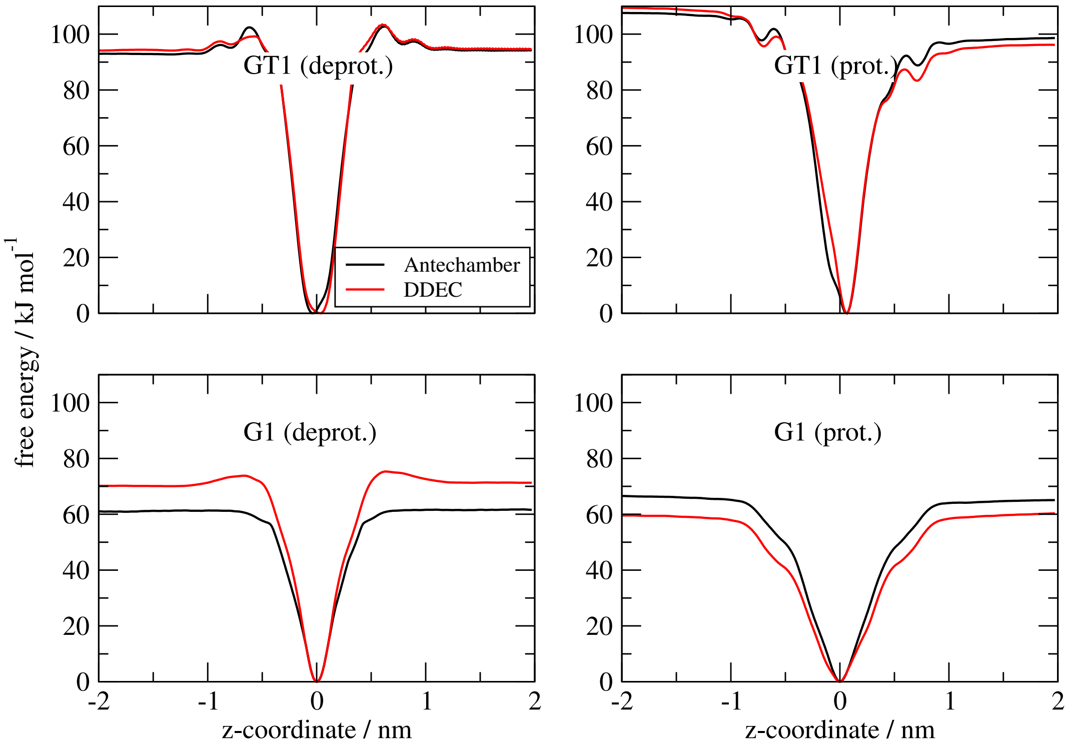

Figure S1: Impact of the partial charge scheme used for parametrization of the ligand: free-energy profiles based on partial charges from the Antechamber program and the DDEC6 approach are depicted in black and red, respectively (see main text for details). Upper row refers to the GT1/CB8 system, lower row to the G1/CB8 system.

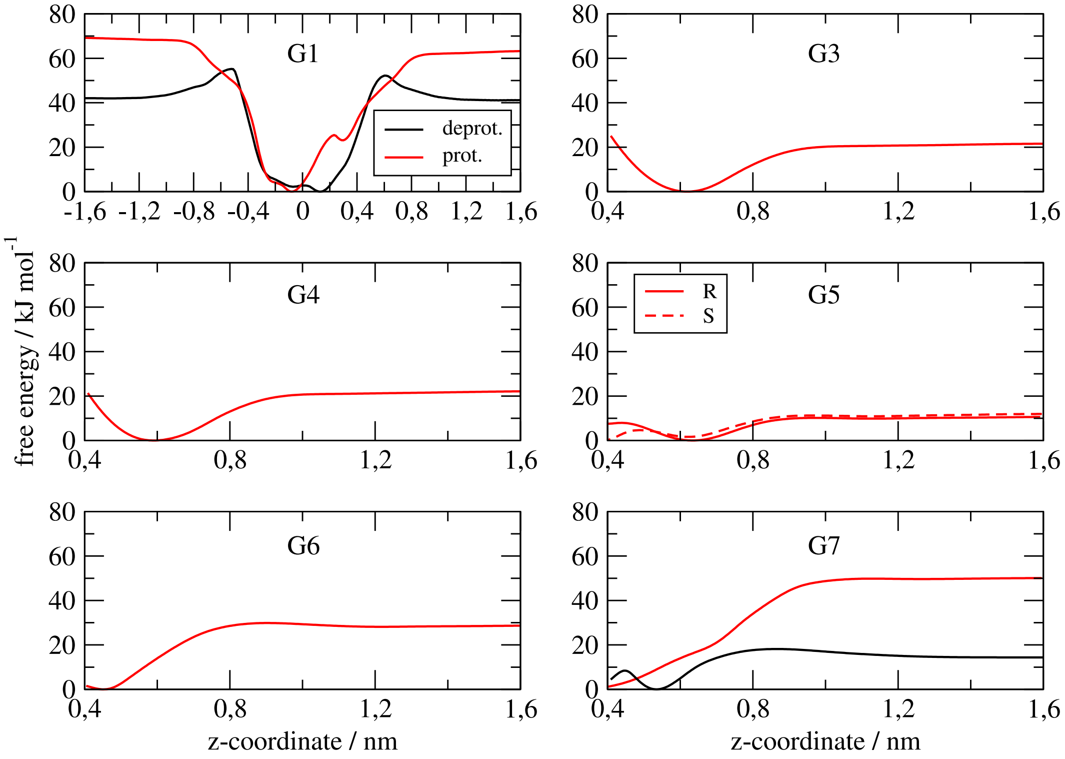

Figure S2: Free-energy profiles for the challenge molecules in protonated (red line) and deprotonated form (black line) binding to the CB7 host. In case of G2 (both species) and the deprotonated forms of G3 to G6, no free-energy profiles could be determined due to the lack of a stable bound state of the complex. For G3 to G7, only one half of the binding path was studied, corresponding to the displacement of the ligand to one side of CB7. The solid (R) and dashed (S) line in case of G5 correspond to the two investigated enantiomeric forms.

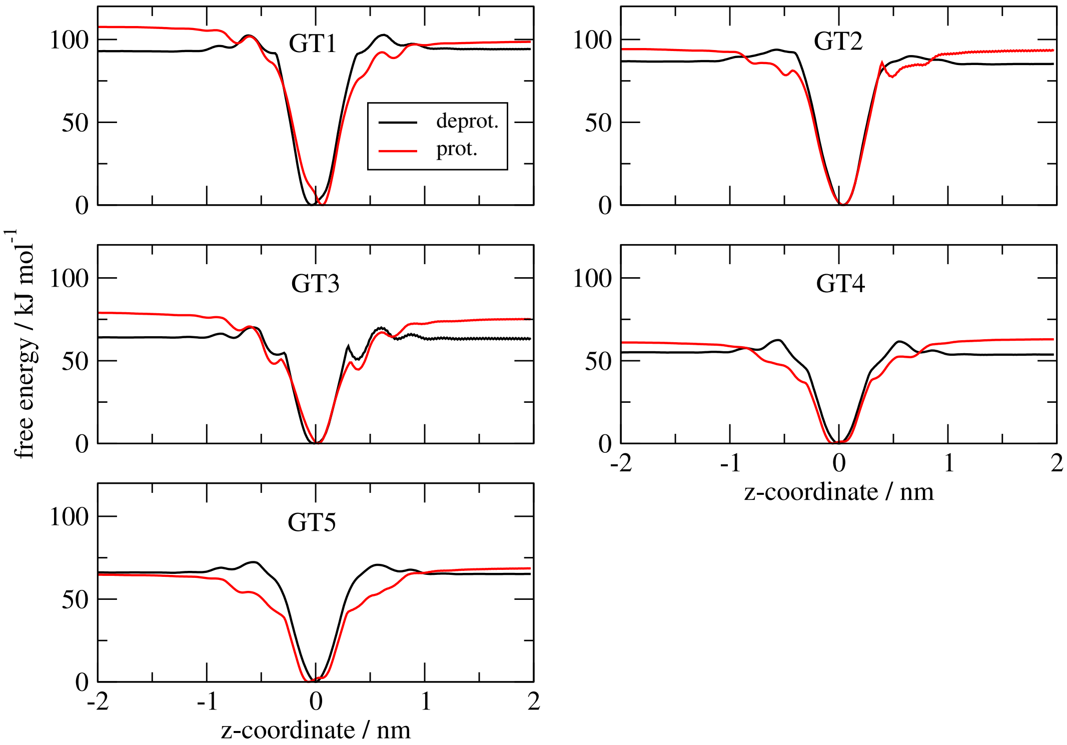

Figure S3: Free-energy profiles for the five training molecules in protonated (red line) and deprotonated (black line) form binding to CB8.

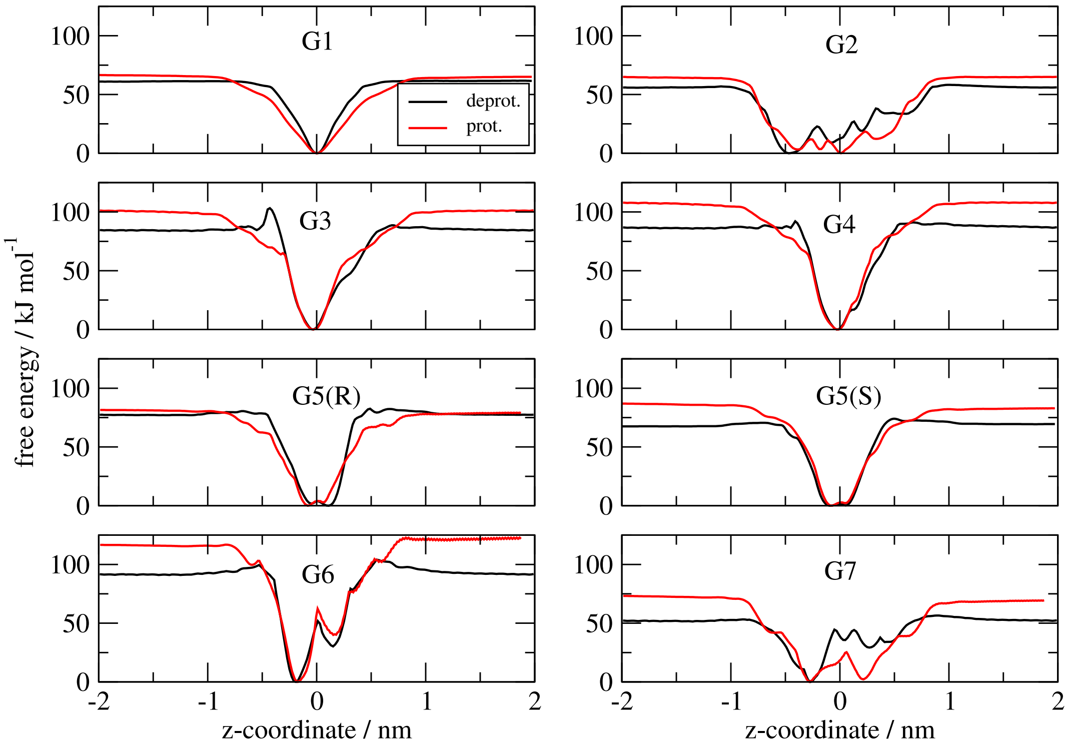

Figure S4: Free-energy profiles for the seven challenge molecules in protonated (red line) and deprotonated (black line) form binding to CB8.

SI2 Tables

Table S1: Parameters of the correction model (Eq. 3 in main text) used for the set of five training molecules.

Molecule	N_{heavy}	$V_{\mathrm{hg}}^{\mathrm{vdW}}$ (kJ mol^{-1})	ΔSASA (Å^2)	TPSA (Å^2)
GT1	13	−38.1	324	3.2
GT2	13	−35.2	346.2	26
GT3	12	−32.3	295.9	46
GT4	8	−25.4	262.4	26
GT5	9	−28.8	277.3	26

Table S2: Parameters of the correction model (Eq. 3 in main text) used for the set of seven challenge molecules.

Molecule	$N_{\mathrm{heavy}}^{\mathrm{eff}}$	$V_{\mathrm{hg}}^{\mathrm{vdW}}$ (kJ mol^{-1})	ΔSASA (Å^2)	TPSA (Å^2)
G1	10.87	−31.9	309.3	12
G2	13.57	−39.9	360.7	23.6
G3	12.31	−36.2	371.9	52.9
G4	12.61	−37.1	372.4	49.8
G5	9.25	−27.2	359.4	29.1
G6	11.29	−33.2	360.4	3.2
G7	11.83	−34.8	361.2	55.8
